# Regimen-Dependent Anti-Cytokine and Immune-Checkpoint Autoantibodies Are Associated with Immunotherapy Toxicity and Response in Melanoma

**DOI:** 10.3390/ijms27219746

**Published:** 2026-10-30

**Authors:** Robin Reschke, Hans-Dieter Zucht, Petra Budde, Joanna Mangana, Reinhard Dummer, Claudia Pföhler, Kilian Wistuba-Hamprecht, Benjamin Weide, Friedegund Meier, Lara-Elena Hakim-Meibodi, Carsten Schulz, Jasmin Richter, Alexander H. Enk, Manuel Bräutigam, Thi Nhat Minh Nguyen, Jessica C. Hassel

**Affiliations:** 1 Department of Dermatology, University Hospital Heidelberg and National Center for Tumor Diseases (NCT), NCT Heidelberg, a Partnership Between DKFZ and University Hospital Heidelberg, 69120 Heidelberg, Germany; 2 German Cancer Consortium (DKTK), Core Center DKFZ Heidelberg, 69120 Heidelberg, Germany; 3 Medical Faculty, University Heidelberg, 69120 Heidelberg, Germany; 4 German Cancer Research Center (DKFZ), Applied Tumor Immunity, 69120 Heidelberg, Germany; 5 Alden Scientific Immune GmbH, 44227 Dortmund, Germany; 6 Department of Dermatology, University Hospital Zürich, 8091 Zürich, Switzerland; 7 Faculty of Medicine, University of Zürich, 8006 Zürich, Switzerland; 8 Department of Dermatology, University Hospital Zürich, University of Zürich, 8091 Zürich, Switzerland; 9 Department of Dermatology, Kantonsspital Aarau, 5001 Aarau, Switzerland; 10 Department of Dermatology, University Hospital Homburg/Saar, 66421 Homburg Saar, Germany; 11 Department of Dermatology, University Hospital Tübingen, 72076 Tübingen, Germany; 12 Skin Cancer Unit, German Cancer Research Center (DKFZ), 69120 Heidelberg, Germany; 13 Department of Dermatology, Venereology and Allergology, University Medical Center Mannheim, 68167 Mannheim, Germany; 14 DKFZ Hector Cancer Institute at the University Medical Center Mannheim, 68167 Mannheim, Germany; 15 Mannheim Institute for Innate Immunoscience (MI3), Medical Faculty Mannheim, University of Heidelberg, 68167 Mannheim, Germany; 16 Skin Cancer Center at the University Cancer Center and National Center for Tumor Diseases Dresden, Department of Dermatology, University Hospital Carl Gustav Carus Dresden, 01307 Dresden, Germany

**Keywords:** melanoma, irAEs, toxicity, response, antibodies, cytokines, checkpoints

## Abstract

Immune checkpoint inhibitor (ICI) efficacy and immune-related adverse events (irAEs) in melanoma are influenced by the pre-treatment immune state. We investigated whether baseline autoantibodies directed against cytokines, chemokines, cytokine receptors, and immune-checkpoint/costimulatory proteins were associated with outcome in a regimen-dependent manner. Baseline sera from patients with metastatic melanoma were profiled on a multiplex bead array of 86 cytokine, chemokine, cytokine-receptor and immune-checkpoint antigens, and associations with best overall response and irAE development were assessed within pre-specified treatment strata by label-permutation testing with Benjamini–Hochberg false-discovery-rate control (SAM as a sensitivity analysis). Associations differed by ICI regimen. Anti-IL17A and anti-CCL2 reactivity was higher in patients developing irAEs under ipilimumab monotherapy (both *p* = 0.003) and shifted in the opposite direction under pembrolizumab without prior CTLA-4 therapy. Higher anti-PD-L1/CD274 and anti-CCL5 and lower anti-KIT reactivity were associated with response to pembrolizumab without prior CTLA-4 therapy (*p* = 0.025, *p* = 0.002, *p* = 0.006), and lower anti-MCAM and anti-CSF3 reactivity with response under ipilimumab/nivolumab (*p* = 0.001 and *p* = 0.002). No association survived false-discovery-rate correction, so all findings are exploratory and hypothesis-generating; higher baseline anti-IL10 reactivity was a leading irAE signal under pembrolizumab without prior CTLA-4 therapy (*p* = 0.027). These regimen-dependent humoral markers warrant prospective, regimen-stratified validation.

## 1. Introduction

Immune checkpoint inhibitors have transformed the treatment of advanced melanoma, yet both therapeutic benefit and immune-related adverse events vary markedly between patients [1,2]. Although tumor-intrinsic determinants contribute to these differences, increasing evidence indicates that the host immune state prior to treatment also shapes subsequent response and toxicity [3,4]. Circulating autoantibodies are attractive markers of this immune set-point because they are stable, readily measurable in serum, and can reflect pre-existing immune dysregulation before initiation of checkpoint blockade [5].

Autoantibodies directed against cytokines and their receptors are particularly relevant because naturally occurring or disease-associated antibodies can alter cytokine bioavailability or function. Neutralizing autoantibodies against interferons or the IL-17-family cytokines provide established examples of biologically consequential anti-cytokine immunity [6,7]. Autoantibodies against immune-checkpoint or costimulatory molecules could likewise mark or potentially modify immune regulatory circuits relevant to ICI activity. Importantly, CTLA-4 and PD-1 blockade release distinct immunological checkpoints and have different toxicity profiles. A baseline humoral feature may therefore have different clinical associations depending on the therapeutic mechanism.

We previously observed that regimen-stratified analyses can uncover autoantibody associations. Here, we extend this approach to a focused panel of cytokines, chemokines, cytokine receptors, and immune-checkpoint/costimulatory proteins. We tested the hypothesis that baseline reactivity to these immune mediators would be associated with response and irAE development in a regimen-dependent manner, with particular emphasis on patterns that change systematically across anti-CTLA-4- and -PD-1-based treatment. The motivation for the present work came from our earlier survey of 832 antigens in this cohort, in which autoantibodies against cytokines and cytokine receptors, including anti-IL4R, were associated with immune-related toxicity and with survival [8]. To build on that observation, we assembled a focused panel enriched for cytokines, chemokines, their receptors and immune-checkpoint proteins and re-measured it in the same patients, so that these candidate associations could be examined at greater depth within individual treatment settings. Samples that could not be re-measured were those with insufficient residual serum volume for a further array run, and the 86 antigens were selected on biological grounds as a focused, pre-specified panel that was fixed before any outcome analysis.

## 2. Results

### 2.1. Anti-IL17A and Anti-CCL2 Reactivity and irAEs Across Regimens

**Figure 1:**
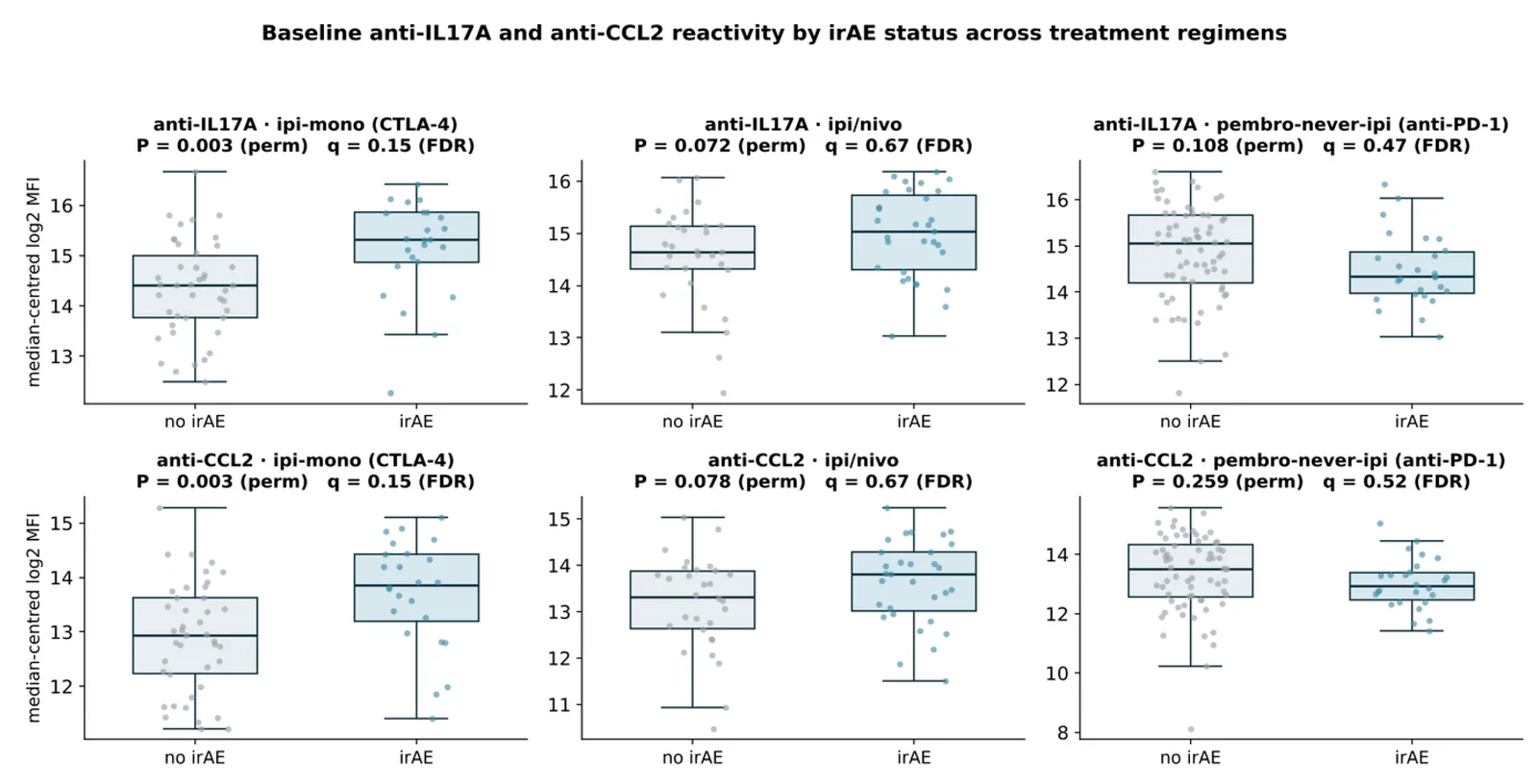
Treatment regimen-dependent association of baseline anti-IL17A and anti-CCL2 reactivity with irAEs. Baseline serum autoantibody reactivity against IL17A (**top row**) and CCL2 (**bottom row**) according to immune-related adverse event (irAE) status in patients treated with ipilimumab monotherapy (**left**), combined ipilimumab/nivolumab (**middle**), or pembrolizumab without prior CTLA-4 therapy (pembro-never-ipi; (**right**)). Anti-IL17A and anti-CCL2 reactivity was higher in patients who developed irAEs during ipilimumab monotherapy (both *p* = 0.003), showed weaker differences in the same direction during ipilimumab/nivolumab treatment (*p* = 0.072 and *p* = 0.078), and shifted toward lower reactivity in patients developing irAEs during pembrolizumab without prior CTLA-4 therapy (*p* = 0.108 and *p* = 0.259). Box plots show medians and interquartile ranges with individual patient values overlaid. *p* values are from two-sided permutation tests. The intermediate and anti-PD-1 comparisons did not reach conventional statistical significance and should therefore be interpreted as a directional cross-regimen pattern rather than evidence of a continuous biological gradient. Group sizes for each comparison were: ipilimumab monotherapy, 42 patients without and 24 with irAEs; ipilimumab/nivolumab, 30 without and 31 with irAEs; and pembrolizumab without prior CTLA-4 therapy, 68 without and 26 with irAEs.

**Figure 2:**
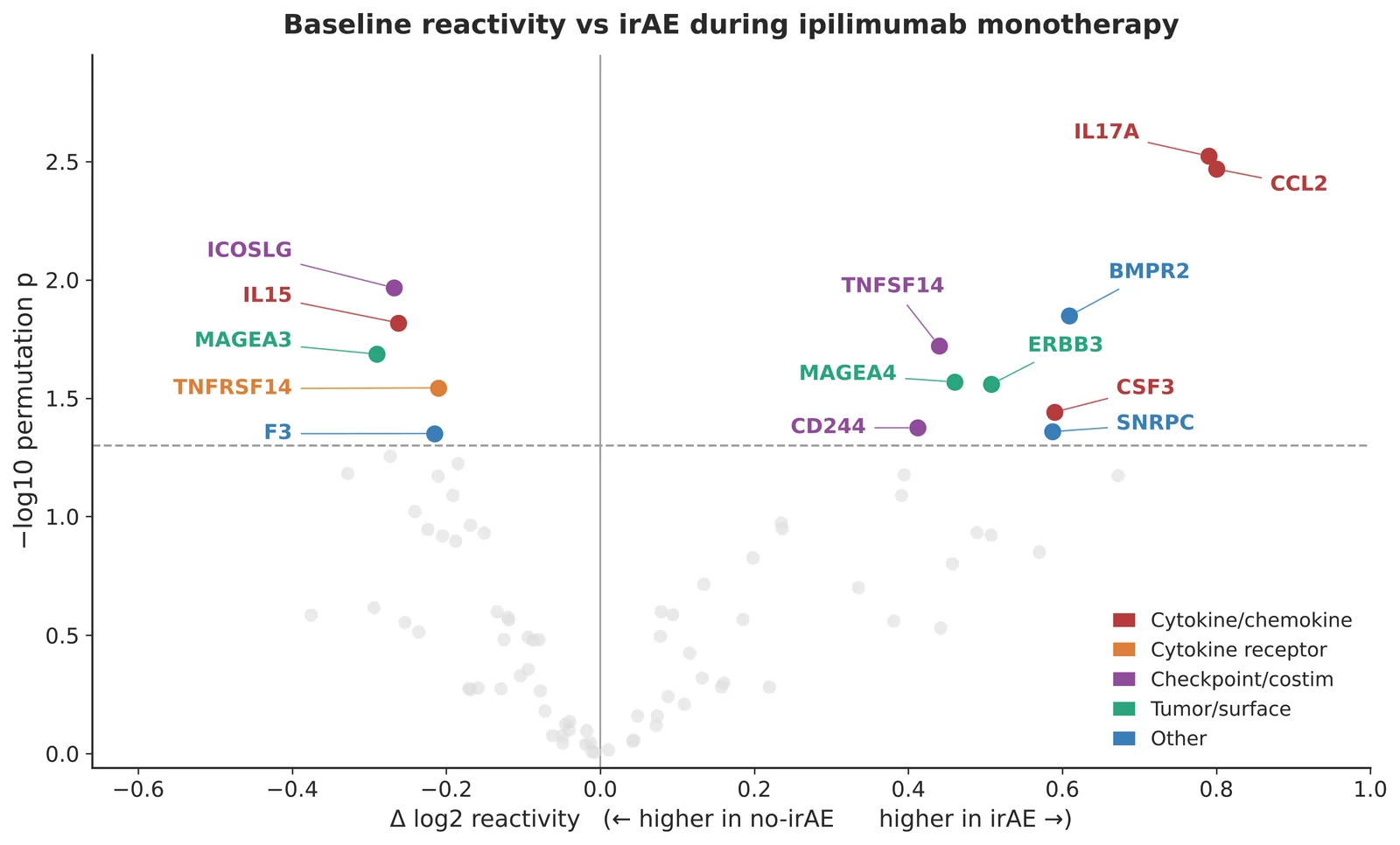
Baseline anti-cytokine and immune-checkpoint autoantibody associations with irAEs during ipilimumab monotherapy. Volcano plot of baseline autoantibody reactivity according to subsequent irAE development in patients receiving ipilimumab monotherapy. Anti-IL17A and anti-CCL2 were among the strongest positive associations with irAE development. The x-axis indicates the difference in log2 reactivity between patients with and without irAEs, with positive values indicating higher reactivity in patients developing irAEs. The y-axis indicates −log10 permutation *p* value. The horizontal dashed line denotes the nominal significance threshold of *p* = 0.05. Colored points denote nominally significant antigens and are classified as cytokine/chemokine, cytokine receptor, checkpoint/costimulatory, tumor/surface, or other; gray points denote the remaining tested antigens. The comparison included 42 patients without and 24 with irAEs.

Baseline antibodies to IL17A and CCL2/MCP-1 were elevated in patients with irAEs under anti-CTLA-4 monotherapy (ipi-monotherapy: both *p* = 0.003; *p* < 0.001 for both in the pooled CTLA-4-based cohort). The same direction was retained but attenuated under combined ipilimumab/nivolumab (anti-IL17A *p* = 0.072; anti-CCL2 *p* = 0.078). In contrast, under pembrolizumab without prior CTLA-4 therapy, both markers shifted in the opposite direction, with lower baseline reactivity among patients who developed irAEs (anti-IL17A *p* = 0.108; anti-CCL2 *p* = 0.259) (Figure 1). Thus, although the intermediate and anti-PD-1 comparisons were not individually significant at the conventional 0.05 threshold, the direction of effect changed systematically across the three treatment settings (Figure 1). In ipilimumab monotherapy, anti-IL17A and anti-CCL2 were among the strongest irAE-associated signals (Figure 2). A weaker, directionally similar pattern was observed for colitis under CTLA-4-based regimens (ipilimumab monotherapy and ipilimumab/nivolumab): higher anti-IL17A reactivity appeared among the positive associations, alongside L1CAM, CD40LG, SNRPA, ERBB3, TNFSF14 and CEACAM1, whereas anti-CCL2 was shifted in the same direction without reaching nominal significance (Supplementary Figure S2). These exploratory data suggest that the IL17A/CCL2 signal may be particularly relevant to CTLA-4-associated toxicity. Formal interaction tests indicated that these associations were stronger under ipilimumab monotherapy than under the other regimens, both for anti-IL17A (interaction odds ratio 2.59, 95% CI 1.30 to 5.17; *p* = 0.007) and for anti-CCL2 (2.23, 1.19 to 4.21; *p* = 0.013). When death was treated as a competing event, higher baseline reactivity remained associated with a greater cause-specific hazard of immune-related toxicity (anti-IL17A hazard ratio 2.05, 1.32 to 3.20; anti-CCL2 1.79, 1.19 to 2.71; Supplementary Figure S9), and anti-IL17A was also nominally higher in patients who developed colitis under CTLA-4-based regimens (permutation *p* = 0.045), with a weaker, non-significant shift for anti-CCL2 (*p* = 0.057).

### 2.2. irAE-Associated Autoantibodies Under Pembrolizumab Without Prior CTLA-4 Therapy

**Figure 3:**
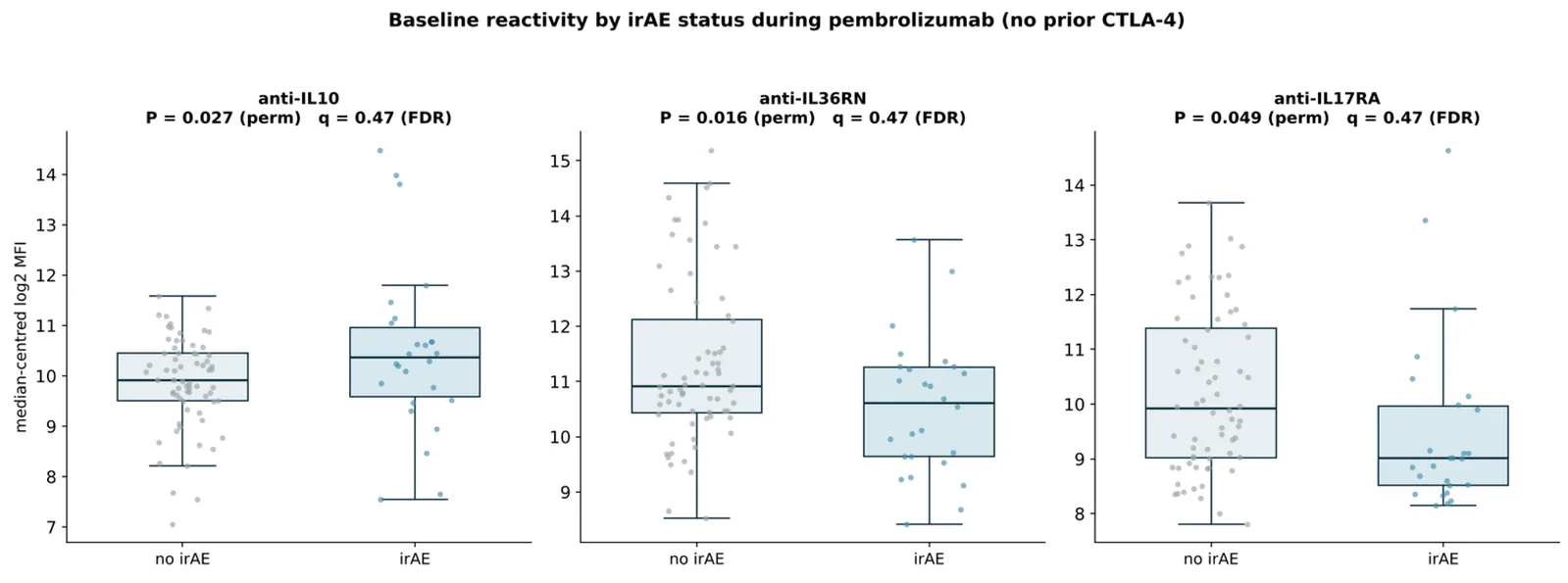
Baseline anti-IL10, anti-IL36RN, and anti-IL17RA reactivity according to irAE development during pembrolizumab without prior CTLA-4 therapy. Baseline autoantibody reactivity against IL10, IL36RN, and IL17RA according to subsequent irAE development in patients receiving pembrolizumab without prior CTLA-4 therapy. Anti-IL10 reactivity was higher in patients developing irAEs (permutation *p* = 0.027; Welch *t*-test *p* = 0.089); it was a leading signal for this endpoint but did not survive Benjamini–Hochberg false-discovery-rate correction and is reported as exploratory. Anti-IL36RN reactivity was lower in patients developing irAEs (*p* = 0.016), as was anti-IL17RA reactivity (*p* = 0.049). Box plots show medians and interquartile ranges with individual patient values overlaid. The comparison included 68 patients without and 26 patients with irAEs.

**Figure 4:**
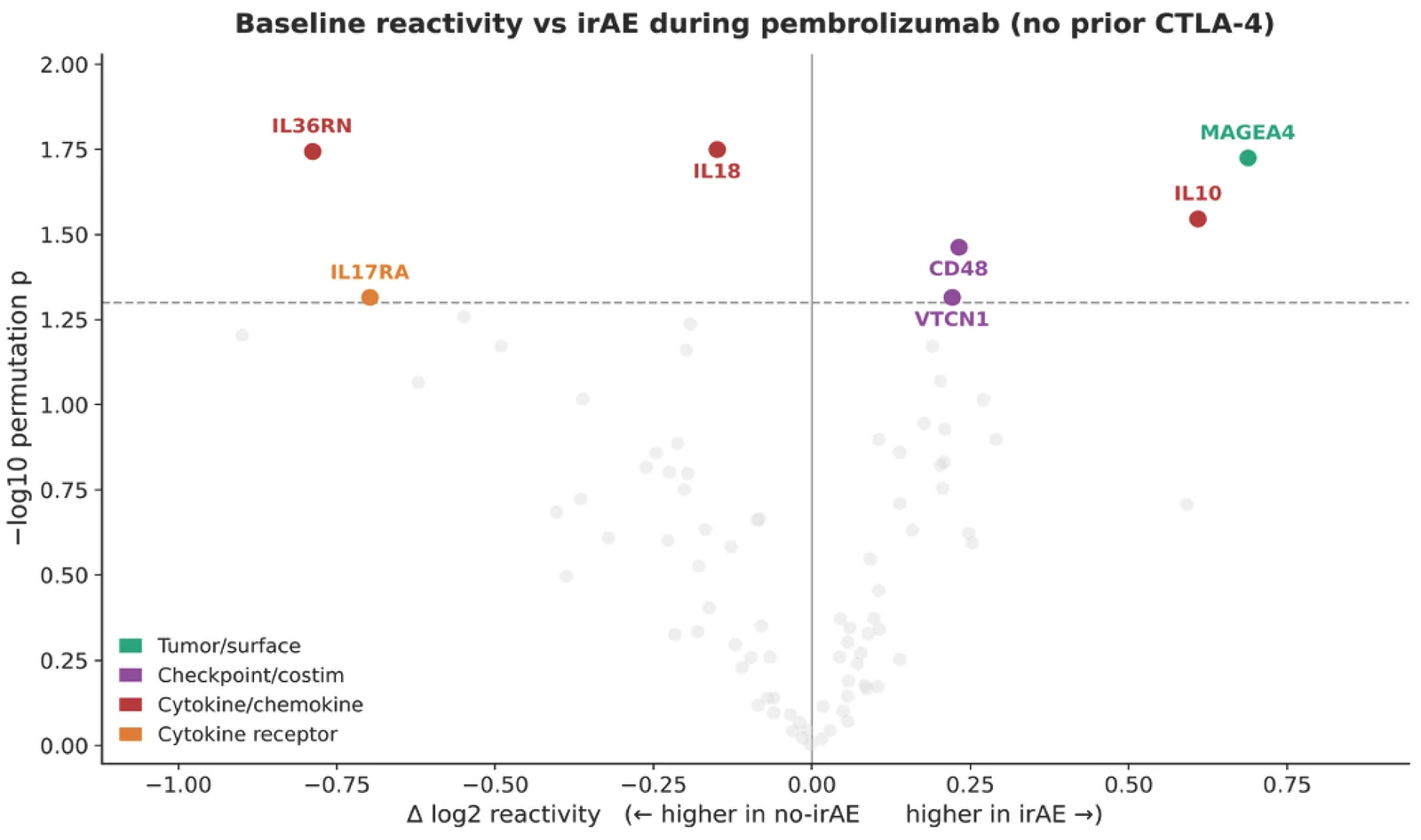
Baseline anti-cytokine and immune-checkpoint autoantibody associations with irAEs during pembrolizumab without prior CTLA-4 therapy. Volcano plot of baseline autoantibody reactivity according to subsequent irAE development in patients receiving pembrolizumab without prior CTLA-4 therapy (pembro-never-ipi). IL10, MAGEA4, and CD48 were shifted toward higher reactivity in patients developing irAEs, whereas IL17RA, IL18, and IL36RN were shifted toward patients without irAEs. The x-axis indicates the difference in log2 reactivity between outcome groups, with positive values indicating higher reactivity in patients developing irAEs. The y-axis indicates −log10 permutation *p* value. The horizontal dashed line denotes the nominal significance threshold of *p* = 0.05. Colored points denote nominally significant antigens according to antigen class; gray points denote the remaining tested antigens. Multiple testing was controlled using the Benjamini–Hochberg procedure across the 86 antigens tested within this treatment group and endpoint; SAM was used only as a sensitivity analysis. The comparison included 68 patients without and 26 with irAEs.

A different pattern emerged in patients receiving pembrolizumab without prior CTLA-4 therapy. Baseline anti-IL10 reactivity was higher in patients who developed irAEs (permutation *p* = 0.027; Figure 3), whereas anti-IL36RN (*p* = 0.016) and anti-IL17RA (*p* = 0.049) reactivity was lower; the parametric Welch *t*-test for anti-IL10 was not significant (*p* = 0.089). In the SAM sensitivity analysis, anti-IL10 ranked third of the 86 antigens for this endpoint, after anti-IL36RN and anti-MAGEA4 (SAM q = 0.22). After Benjamini-Hochberg correction for the 86 antigens tested for this endpoint in this treatment group, no antigen reached q < 0.05; anti-IL10 is therefore reported as a leading nominal association that requires independent validation. Several further analyses were consistent with this association. A formal interaction test did not establish regimen specificity for anti-IL10 (interaction odds ratio 1.56, 95% CI 0.95 to 2.55; *p* = 0.077), but the association persisted when death was treated as a competing event (cause-specific hazard ratio 1.50, 95% CI 1.02 to 2.21; Supplementary Figure S9A) and after adjustment for serum LDH and centre (odds ratio 3.39, 95% CI 1.33 to 8.66), with a similar estimate after additional adjustment for performance status (odds ratio 3.42, 95% CI 1.33 to 8.78). Interaction testing did support regimen specificity for anti-IL36RN (interaction odds ratio 0.60, 95% CI 0.39 to 0.92; *p* = 0.020) and anti-IL17RA (interaction odds ratio 0.60, 95% CI 0.39 to 0.91; *p* = 0.016). Among the response markers, the interaction was significant for anti-CCL5 (interaction odds ratio 1.51; *p* = 0.009) and anti-KIT (0.59; *p* = 0.009) but not for anti-PD-L1/CD274 (interaction odds ratio 1.94, 95% CI 0.88 to 4.28; *p* = 0.099), which is reported as a nominal association. Anti-IL10 reactivity was also higher in the 11 patients who developed grade 3 or 4 irAEs than in the 50 patients without grade 3 or 4 events (mean difference 1.28 log2 units, Welch *t*-test *p* = 0.016), whereas the ipilimumab-monotherapy groups had too few grade 3 or 4 events to support a separate severity analysis. Volcano analyses of the broader pembrolizumab cohorts were consistent with treatment-history-dependent heterogeneity. In the analysis restricted to patients without prior CTLA-4 therapy, antibodies against IL10, MAGEA4, and CD48 were among the positive irAE-associated signals, whereas anti-IL17RA, -IL18, and -IL36RN were shifted toward the no-irAE group (Figure 4). When all pembrolizumab-treated patients were considered, additional nominal associations included higher MAGEA4, ERBB2, and IL6 reactivity in irAE cases and higher IL17A, IL17RA, IL22RA2, and SNRPC reactivity in patients without irAEs (Supplementary Figure S3). Because prior anti-CTLA-4 exposure may alter the immune set-point, the group without prior CTLA-4 therapy was retained as the principal anti-PD-1 analysis.

### 2.3. Autoantibodies Associated with Treatment Response

**Figure 5:**
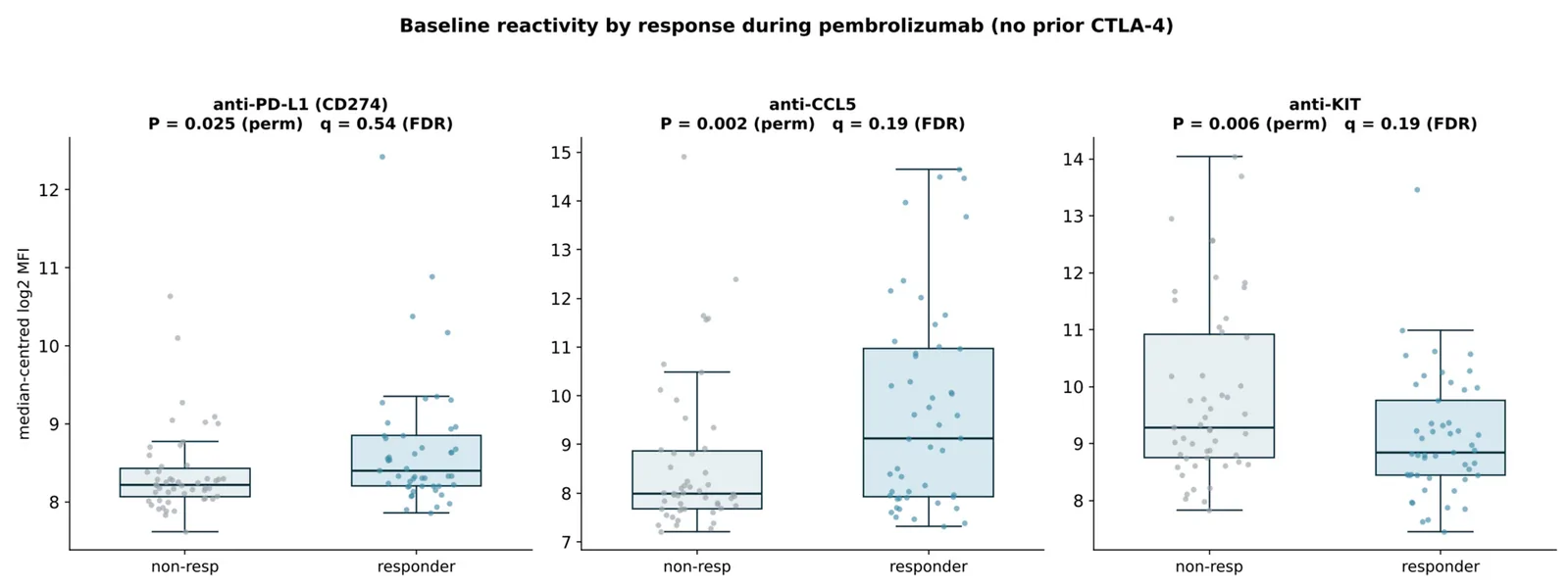
Baseline anti-PD-L1, anti-CCL5, and anti-KIT reactivity is associated with response to pembrolizumab without prior CTLA-4 therapy. Baseline autoantibody reactivity against PD-L1/CD274, CCL5, and KIT according to best overall response in patients receiving pembrolizumab without prior CTLA-4 therapy. Responders showed higher anti-PD-L1/CD274 and anti-CCL5 reactivity and lower anti-KIT reactivity than non-responders (*p* = 0.025, *p* = 0.002, and *p* = 0.006, respectively). Box plots show medians and interquartile ranges with individual patient values overlaid. The comparison included 51 non-responders and 49 responders.

**Figure 6:**
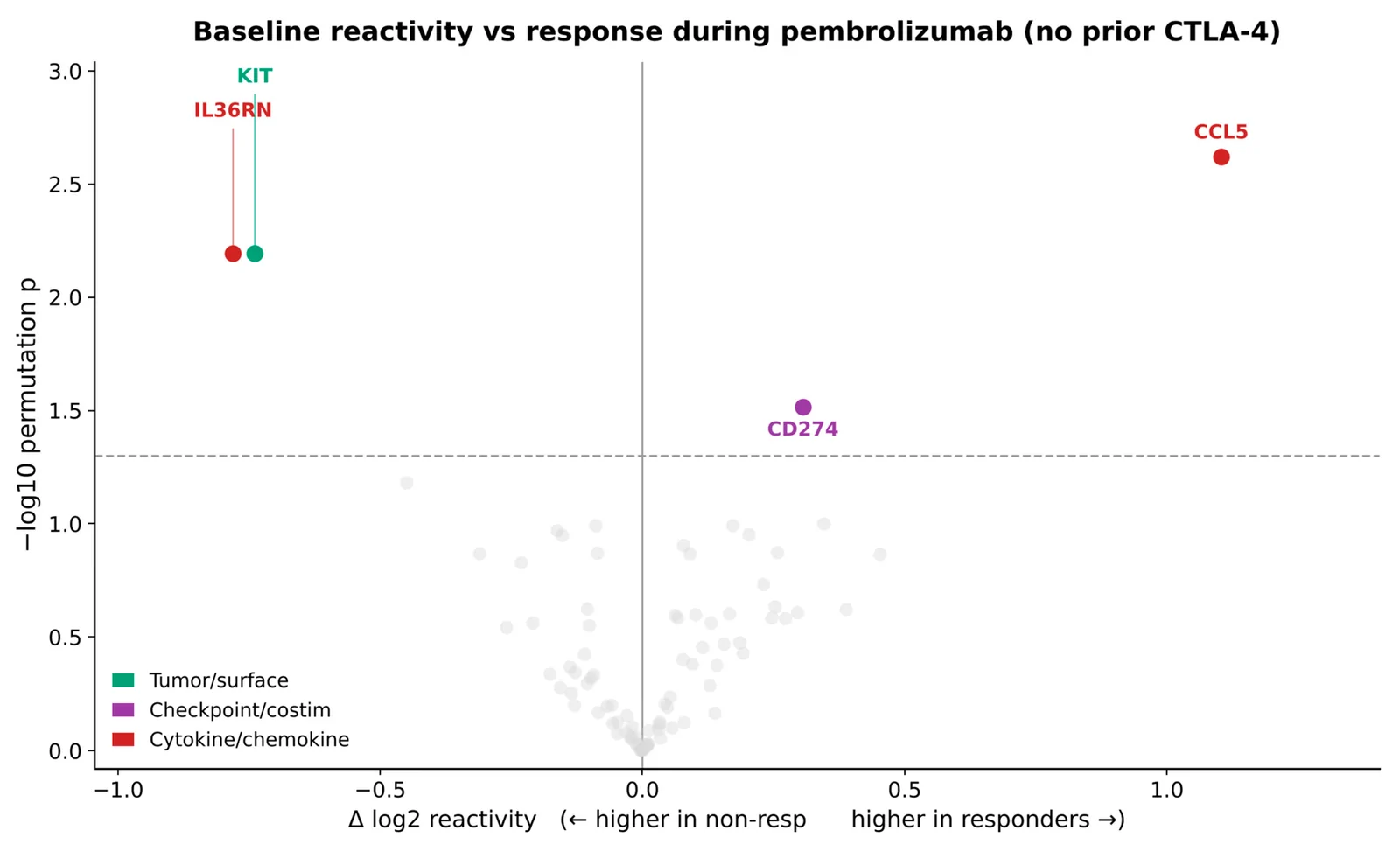
Baseline anti-cytokine and immune-checkpoint autoantibody associations with response to pembrolizumab without prior CTLA-4 therapy. Volcano plot of baseline autoantibody reactivity according to best overall response in patients receiving pembrolizumab without prior CTLA-4 therapy. CCL5 and PD-L1/CD274 were shifted toward higher reactivity in responders, whereas IL36RN and KIT were shifted toward non-response. The x-axis indicates the difference in log2 reactivity between responders and non-responders, with positive values indicating higher reactivity in responders. The y-axis indicates −log10 permutation *p* value. The horizontal dashed line denotes the nominal significance threshold of *p* = 0.05. Colored points denote nominally significant antigens according to antigen class; gray points denote the remaining tested antigens. The comparison included 51 non-responders and 49 responders.

Within the group receiving pembrolizumab without prior CTLA-4 therapy, responders had higher baseline anti-PD-L1/CD274 and anti-CCL5 reactivity and lower anti-KIT reactivity than non-responders (*p* = 0.025, *p* = 0.002, and *p* = 0.006, respectively; Figure 5). The corresponding volcano analysis placed CCL5 and CD274 on the responder-associated side, while anti-IL36RN and -KIT were shifted toward non-response (Figure 6). The association between pre-existing anti-PD-L1 reactivity and response is mechanistically notable because the antibody target lies directly within the pathway engaged by anti-PD-1 therapy; however, the current binding assay does not establish whether these antibodies alter PD-L1 function. Analysis of all pembrolizumab-treated patients showed a broadly compatible but not identical pattern, with anti-CD274 remaining responder-associated while anti-TNF, -IL36RN, -KIT, and a reference autoantigen were shifted toward non-response (Supplementary Figure S4). These differences further support separating anti-PD-1 treatment without prior CTLA-4 therapy from anti-PD-1 therapy administered after prior anti-CTLA-4 exposure.

**Figure 7:**
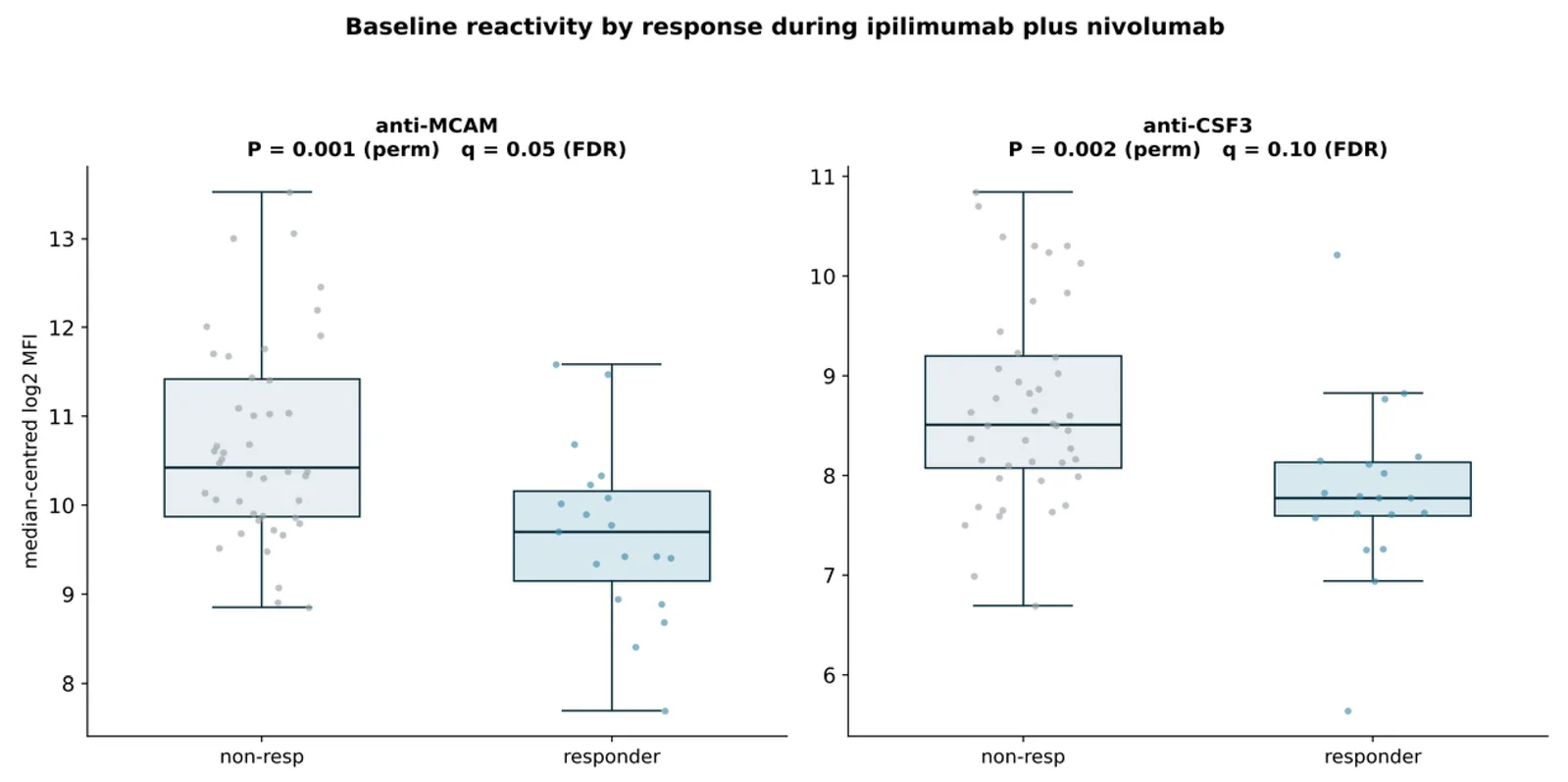
Baseline anti-MCAM and anti-CSF3 reactivity is lower in responders to combined ipilimumab/nivolumab. Baseline anti-MCAM and anti-CSF3/G-CSF autoantibody reactivity according to best overall response in patients treated with combined ipilimumab/nivolumab. Responders showed lower anti-MCAM and anti-CSF3 reactivity than non-responders (*p* = 0.001 and *p* = 0.002, respectively). Box plots show medians and interquartile ranges with individual patient values overlaid. The comparison included 44 non-responders and 19 responders.

In the ipilimumab/nivolumab group, response was associated with lower baseline anti-MCAM and anti-CSF3/G-CSF reactivity (*p* = 0.001 and *p* = 0.002, respectively; Figure 7). The volcano analysis likewise placed anti-MCAM and anti-CSF3 among the strongest non-responder-associated signals, while anti-IL21R showed a nominal shift toward responders (Supplementary Figure S5). No comparably strong irAE signal emerged in the ipilimumab/nivolumab volcano analysis (Supplementary Figure S6), consistent with the weaker intermediate IL17A/CCL2 differences observed in the cross-regimen comparison. The ipilimumab-monotherapy response analysis showed a broader set of nominal associations than the other treatment strata. Higher baseline reactivity to CSF2, CSF3, IL23A, IL6R, IL22RA2, MCAM, and a reference autoantigen was observed among responders, whereas several checkpoint/costimulatory and inflammatory targets including CD74, CD40, HAVCR2, CD86, VSIR, TNFRSF14, CXCL8, EGFR, and ERBB2 were shifted toward non-response (Supplementary Figure S7). These findings did not define a cross-validated classifier and are therefore best viewed as a map of candidate regimen-specific immune states rather than a predictive signature. In formal interaction tests, the response association differed by treatment regimen for anti-MCAM under combined ipilimumab and nivolumab (interaction odds ratio 0.28, 95% CI 0.13 to 0.59; *p* = 0.001) and for anti-CSF3 under ipilimumab monotherapy (2.08, 1.15 to 3.75; *p* = 0.015).

### 2.4. Autoantibodies and Overall Survival

**Figure 8:**
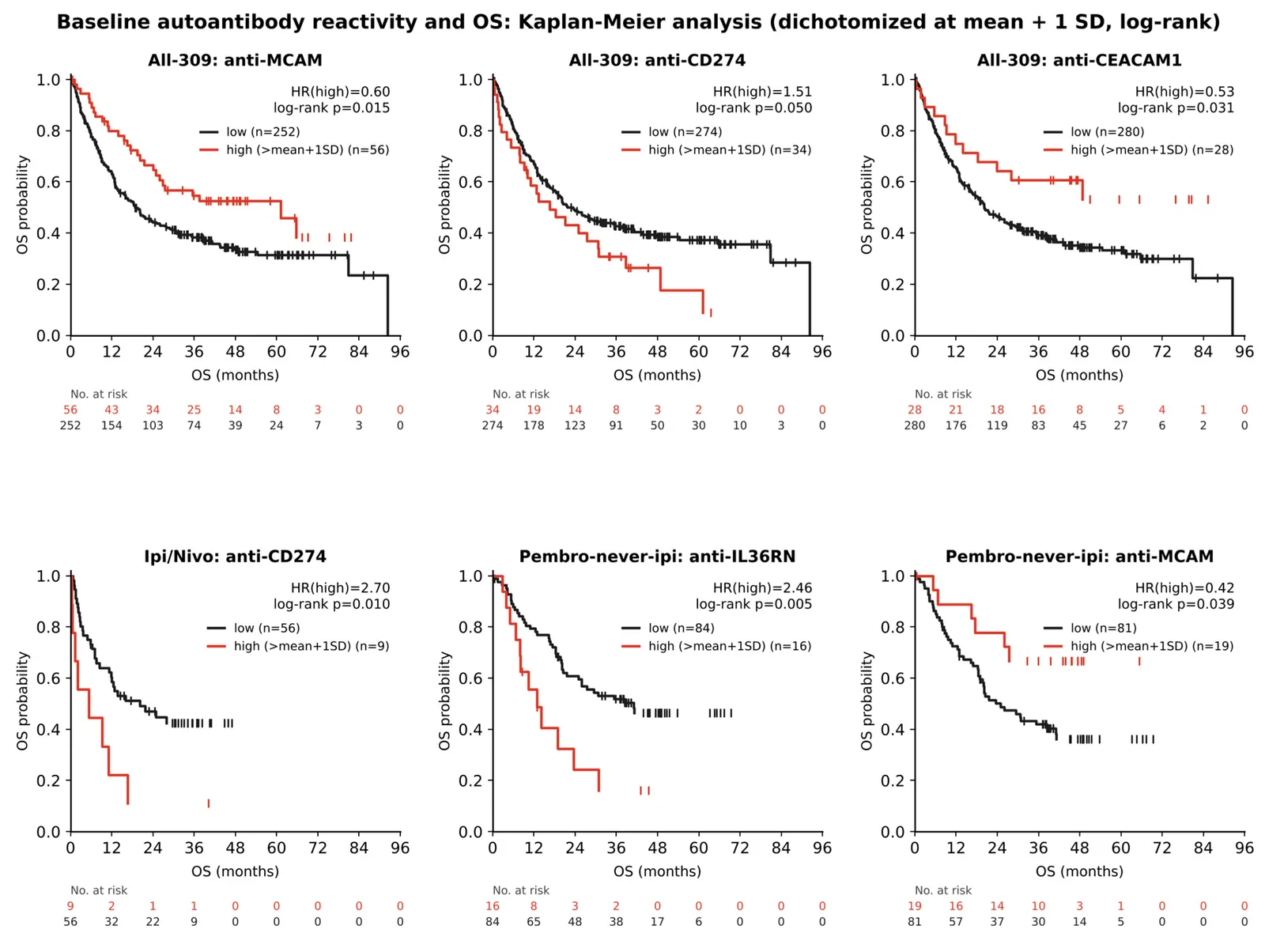
Baseline autoantibody reactivity and overall survival in melanoma patients receiving immune checkpoint inhibitors. Kaplan–Meier curves showing overall survival (OS) according to baseline anti-MCAM, anti-PD-L1/CD274, and anti-CEACAM1 reactivity in the pooled cohort ((**top row)**, left to right), anti-CD274 reactivity in patients receiving ipilimumab/nivolumab (**bottom left**), and anti-IL36RN and anti-MCAM reactivity in patients receiving pembrolizumab without prior CTLA-4 therapy (**bottom middle and right**). Patients were dichotomized into high reactivity (>mean + 1 standard deviation; red) and low reactivity (≤mean + 1 standard deviation; black). Hazard ratios (HRs) compare the high- with the low-reactivity group; *p* values are from log-rank tests. Tick marks indicate censored observations, and numbers at risk are shown below each panel. Analyses are exploratory, and displayed *p* values are nominal. Numbers of patients and deaths per panel were: pooled anti-MCAM, 56 high-reactivity (27 deaths) versus 252 low-reactivity (161 deaths); pooled anti-PD-L1/CD274, 34 (26 deaths) versus 274 (162 deaths); pooled anti-CEACAM1, 28 (12 deaths) versus 280 (176 deaths); ipilimumab/nivolumab anti-CD274, 9 (8 deaths) versus 56 (31 deaths); pembrolizumab without prior CTLA-4 therapy, anti-IL36RN, 16 (12 deaths) versus 84 (42 deaths); and pembrolizumab without prior CTLA-4 therapy, anti-MCAM, 19 (6 deaths) versus 81 (48 deaths).

Exploratory Kaplan–Meier analyses further identified associations between baseline autoantibody reactivity and overall survival (OS; Figure 8). In the pooled cohort, high anti-MCAM and anti-CEACAM1 reactivity was associated with longer OS (HR = 0.60, log-rank *p* = 0.015 and HR = 0.53, *p* = 0.031, respectively), whereas high anti-PD-L1/CD274 reactivity showed a borderline association with shorter OS (HR = 1.51, *p* = 0.050). In treatment-specific analyses, high anti-CD274 reactivity was associated with shorter OS during ipilimumab/nivolumab therapy (HR = 2.70, *p* = 0.010). Among patients receiving pembrolizumab without prior CTLA-4 therapy, high anti-IL36RN reactivity was associated with shorter OS (HR = 2.46, *p* = 0.005), whereas high anti-MCAM reactivity was associated with longer OS (HR = 0.42, *p* = 0.039). These findings represent nominal exploratory associations requiring independent validation. Modelling each antibody as a continuous variable gave concordant results. Higher anti-MCAM reactivity was associated with longer overall survival (hazard ratio 0.87 per log2 unit, 95% CI 0.79 to 0.96), higher anti-CEACAM1 reactivity with longer progression-free survival (0.85, 0.74 to 0.97), and, among patients receiving pembrolizumab without prior CTLA-4 therapy, higher anti-IL36RN reactivity with shorter overall and progression-free survival (1.29, 1.08 to 1.53 and 1.26, 1.08 to 1.46). The high-reactivity cut-point was derived from the pooled cohort, and the number of patients, events and comparisons underlying each curve is reported in Supplementary Table S2. The proportional-hazards assumption held for all displayed markers (Schoenfeld-residual *p* > 0.2), and the anti-MCAM overall-survival association persisted after adjustment for treatment regimen (adjusted hazard ratio 0.89 per log2 unit, 95% CI 0.80 to 0.98), whereas the anti-CEACAM1 association attenuated (0.93, 0.80 to 1.08) and is therefore partly explained by differences between regimens.

## 3. Discussion

This exploratory study suggests that treatment regimen influences the relationship between baseline anti-cytokine/immune-checkpoint autoantibodies and clinical outcome during ICI therapy. Rather than observing a universal humoral biomarker across checkpoint inhibitors, we found distinct patterns under anti-CTLA-4 monotherapy, combined CTLA-4/PD-1 blockade, and PD-1 blockade without prior CTLA-4 therapy. The clearest cross-regimen pattern involved anti-IL17A and anti-CCL2: reactivity was higher before irAEs under ipilimumab, weaker in the same direction under ipilimumab/nivolumab, and shifted toward lower levels in irAE cases under pembrolizumab without prior CTLA-4 therapy. This directional pattern should not be interpreted as proof of a continuous biological gradient, because the intermediate and anti-PD-1 contrasts were not individually significant. Nevertheless, it provides a coherent, testable interaction hypothesis that would have been obscured by pooling treatment regimens [5,8,9].

The CTLA-4-associated IL17A/CCL2 pattern is biologically plausible. IL-17-family signaling promotes tissue inflammation and neutrophil recruitment, whereas CCL2 coordinates monocyte trafficking and myeloid inflammation [10,11,12]. Baseline antibodies against these mediators could therefore reflect an immune state predisposed to inflammatory toxicity, or, depending on their epitope and function, could themselves alter cytokine signaling [6,7,13]. The observation that anti-IL17A, and to a lesser extent anti-CCL2, was also shifted toward higher reactivity in the CTLA-4-based colitis analysis supports the rationale for organ-specific validation. However, because the assay quantifies binding rather than neutralizing activity, the present data cannot distinguish functional autoantibodies from biomarkers of a broader loss of immune tolerance [7]. Notably, Váraljai et al. demonstrated that elevated baseline IL-17 signaling was associated with improved clinical outcomes following combined CTLA-4 and PD-1 blockade in melanoma, highlighting the potential dual role of IL-17-mediated immunity in antitumor responses and inflammatory toxicity [14].

PD-1 blockade without prior CTLA-4 therapy was characterized by a distinct set of associations. In this group, higher baseline anti-IL10 reactivity was a leading irAE-associated signal (permutation *p* = 0.027), although it did not survive Benjamini–Hochberg correction and is reported as exploratory. IL-10 is a central regulator of immune homeostasis, limiting excessive inflammatory responses and tissue damage. Importantly, naturally occurring anti-IL-10 autoantibodies have been described in humans, and functional neutralization of IL-10 has recently been linked to exaggerated inflammatory responses, providing biological precedent for the possibility that anti-IL10 reactivity could impair immune counter-regulation [15,16,17,18]. The simultaneous lower anti-IL36RN reactivity and lower anti-IL17RA reactivity among patients with irAEs further suggests that anti-PD-1 toxicity may involve a humoral configuration distinct from that observed under CTLA-4 blockade. The direction of the anti-IL36RN associations is compatible with a potential link between inflammatory toxicity and therapeutic benefit: lower baseline reactivity was associated with irAE development, whereas high reactivity was associated with shorter OS. However, these separate exploratory associations do not establish a direct relationship between irAE occurrence and survival in this cohort. Anti-IL10 reached nominal significance under permutation testing and ranked third of 86 antigens in the SAM sensitivity analysis (SAM q = 0.22), but it did not survive Benjamini–Hochberg correction for the 86 antigens tested for this endpoint in this treatment group and was not significant under the Welch *t*-test. A formal interaction test did not confirm that the association is specific to anti-PD-1 therapy without prior CTLA-4 therapy, so the finding is presented as a toxicity-associated signal that persisted after competing-risk and covariate adjustment but whose regimen specificity remains to be established. Among the response markers, higher baseline anti-CCL5 reactivity was additionally associated with longer progression-free survival in this group (hazard ratio 0.86 per log2 unit, 95% CI 0.75 to 0.98), consistent with its association with response.

The response analyses likewise emphasize regimen specificity. Under pembrolizumab without prior CTLA-4 therapy, higher anti-PD-L1/CD274 and anti-CCL5 reactivity and lower anti-KIT reactivity were associated with response. Anti-PD-L1 is of interest because it targets the ligand of the pathway therapeutically disrupted by pembrolizumab, although this was one of the weaker associations, without a significant interaction test, and requires confirmation. Depending on epitope and functional properties, endogenous anti-PD-L1 antibodies could theoretically alter PD-1/PD-L1 signaling or could mark pre-existing immune activation. CCL5 is associated with CD8^+^ T-cell recruitment and the T-cell-inflamed phenotype in melanoma, providing a plausible connection between anti-CCL5 reactivity and the pre-existing immune state relevant to checkpoint-blockade sensitivity [19,20,21]. In contrast, response under ipilimumab/nivolumab was associated with lower anti-MCAM and anti-CSF3, again arguing against a single autoantibody response signature across ICI regimens. A possible methodological implication is that prior treatment history may need to be treated as a biological variable rather than merely a clinical covariate in humoral biomarker studies. The differences between the analyses restricted to patients without prior CTLA-4 therapy and those including all pembrolizumab-treated patients illustrate how previous CTLA-4 exposure can plausibly reshape the baseline state subsequently measured before PD-1 therapy [22].

Several limitations require emphasis. This is a single-cohort discovery study with multiple antigens, endpoints and treatment strata; most associations are nominal, and no association survived Benjamini-Hochberg false-discovery-rate correction, so all findings are reported as exploratory and hypothesis-generating. Subgroup sample sizes limit precision, particularly for organ-specific toxicity and treatment-history analyses. The assay measures binding reactivity and provides no information on neutralization, agonism, IgG subclass, affinity or epitope specificity (total IgG was measured); the associations reported here are biomarker associations and do not establish that these antibodies neutralise or otherwise modulate their targets in vivo, and orthogonal binding confirmation and functional testing of the leading candidates are the natural next steps. A strength of the study is its multicentre composition, which broadens the clinical and demographic range of the cohort and lends the associations greater generalisability than a single-centre series would afford. This design does, however, introduce structure that warrants caution: because the treatment groups were drawn from different centres, with ipilimumab monotherapy contributed by two sites, and because the groups differed in age, centre and age are partly aligned with treatment and could contribute to the between-group differences. Formal interaction testing supported regimen dependence for most of the highlighted antibodies, but because treatment regimen, centre, age and assay plate are partly aligned, interaction tests cannot separate these factors, and confounding by centre or age cannot be excluded; validation in an independent cohort is therefore required. After adjustment for assay plate, the leading associations kept their direction, and only anti-IL36RN and anti-IL17RA irAE associations no longer reached nominal significance. Per-sample median centring expresses each antigen relative to the overall reactivity of the same sample. If overall antibody reactivity differs systematically between groups, this normalisation may influence the comparisons, and the reported associations should therefore be read as differences in relative rather than absolute reactivity.

Ascertainment of immune-related events and the duration of follow-up also varied between regimens, and irAE status was unknown for 25 patients, which could bias the comparison between affected and unaffected patients; the competing-risk analysis was added in part to address this. Prognostic covariates were incompletely recorded, with serum LDH available for about 62 percent and performance status for about 64 percent of patients, which limited adjusted modelling. In the group receiving pembrolizumab without prior CTLA-4 therapy, the anti-IL10 association was essentially unchanged after adjustment for serum LDH, performance status and centre (adjusted odds ratio 3.42, 95% CI 1.33 to 8.78), whereas the ipilimumab-monotherapy signals for anti-IL17A and anti-CCL2 had too few covariate-complete cases to support adjustment.

Brain-metastasis status and baseline immunosuppression were not recorded and could not be considered, and treatment regimen dependence was supported by formal interaction testing for most, though not all, of the highlighted antibodies, on the basis of unadjusted, exploratory *p*-values. Discrimination of the individual markers was modest, with areas under the curve (AUC) of approximately 0.6 to 0.76 and wide confidence intervals (Supplementary Table S5 and Supplementary Figure S8); the panel is therefore not yet a clinical predictor, and claims of biomarker utility are correspondingly moderated. Bootstrap resampling indicated that the ipilimumab-monotherapy anti-IL17A and anti-CCL2 associations and the ipilimumab/nivolumab anti-MCAM and anti-CSF3 response associations were the most stable, whereas the anti-IL10 irAE signal was less stable, in keeping with its borderline significance; external validation in an independent cohort remains essential.

The next logical step for future research would be prospective, regimen-stratified validation using pre-specified antigens and endpoints, ideally accompanied by functional characterization of anti-IL10, anti-IL17A, anti-CCL2, and anti-PD-L1 antibodies. Such studies should determine whether the observed reactivities are mechanistically active or instead serve as stable serum markers of distinct pre-treatment immune states.

## 4. Materials and Methods

### 4.1. Study Population & Stratification

Baseline sera were from patients with metastatic melanoma. Treatment regimens were: ipilimumab monotherapy (*n* = 76), ipilimumab/nivolumab (*n* = 65), and pembrolizumab without prior CTLA-4 therapy (pembrolizumab-never-ipilimumab; *n* = 100; identified via the documented absence of prior CTLA-4 inhibitor therapy). The pembrolizumab-after-ipilimumab group (*n* = 49, a checkpoint-inhibitor follow-up population) was substantially smaller and, because it represents a later-line, checkpoint-inhibitor-experienced population whose prior exposure can confound baseline associations, was not used for the primary analysis and was retained only for selected exploratory comparisons. Clinical information is listed in Table 1. A fifth treatment regimen, nivolumab monotherapy (*n* = 19), completes the analysis cohort of 309 patients and is presented as a separate column in Table 1. Because assignment to this group rests on the documented absence of prior CTLA-4 therapy, this group is described throughout as pembrolizumab without prior CTLA-4 therapy, and systemic therapy other than CTLA-4 blockade was not formally excluded. Each patient is counted in a single treatment group, defined at the first sampled regimen, and a patient flow diagram is provided (Supplementary Figure S1). This was a retrospective, multicentre analysis of patients with metastatic melanoma treated at the participating centres. Patients received their index immunotherapy between September 2011 and July 2018. Baseline serum was drawn on the morning of the first treatment day, before the first dose, which was administered later the same day. Within this group, prior CTLA-4 therapy was absent in all 100 patients by definition; pembrolizumab was documented as the first systemic therapy in 53, prior systemic therapy in 14 (most often BRAF/MEK inhibition or chemotherapy), and prior anti-PD-1 exposure in 2 (one prior PD-1 inhibitor and one adjuvant nivolumab), with pretreatment status not documented in the remainder. The group was therefore defined by the documented absence of prior CTLA-4 therapy rather than by treatment-naivety, and the complete per-patient treatment history is provided in Supplementary Table S3. The analysed patients are a subset of the cohort profiled previously on a broader panel of 832 antigens (ref [8]). Details on the 309 analysed patient samples are provided in Supplementary Figure S1. All analyses were complete-case within each endpoint, with no imputation of missing values. Detailed selection criteria are as described for the source cohort (ref [8]).

### 4.2. Autoantibody Profiling

Serum autoantibody reactivity was measured using a multiplex bead-based platform (ImmunoINSIGHTS; xMAP/Luminex). The analyzed panel comprised 86 antigens spanning cytokines and chemokines, cytokine receptors, immune-checkpoint and costimulatory molecules, selected tumor/surface proteins, reference autoantigens, and technical-control probes. Representative immune targets included IL17A, IL10, CCL2, CCL5, CSF2, CSF3, IL23A, IL6, TNF, IL36RN, IL6R, IL17RA, IL22RA2, PD-L1/CD274, CD27, CD40/CD40LG, ICOSLG, and HAVCR2. Reactivity was expressed as log2 median fluorescence intensity and sample-median centered. The complete antigen list, giving each antigen and its source together with the control beads, is provided in Supplementary Table S1. Antigens were either produced in-house or purchased from third-party vendors: in-house human protein antigens were expressed in Escherichia coli with an N-terminal hexahistidine tag, affinity-purified under denaturing conditions and verified by mass spectrometry, while the remaining recombinant antigens were obtained from commercial suppliers (Supplementary Table S1). Each antigen was covalently coupled to spectrally distinct magnetic microspheres (MagPlex, Luminex Corporation, Austin, TX, USA) by carbodiimide chemistry on a semi-automated liquid-handling system. The panel also carried technical-control beads, namely human IgG, IgA and IgM isotype controls at defined concentrations, bovine serum albumin (BSA) and an Escherichia coli lysate, together with four reference autoantigens (SNRPA, TROVE2/Ro60, SNRPC and CENPH). Serum was diluted 1:100 in assay buffer and pre-adsorbed against an E. coli extract to suppress anti-E. coli reactivity; after incubation for 20 h at 4 to 8 degrees Celsius, bound IgG was detected with an R-phycoerythrin-conjugated anti-human IgG antibody and the beads were read on a FlexMap3D analyser, with reactivity recorded as median fluorescence intensity (MFI). As a quality-control step, bead identities measured on fewer than 30 beads were excluded. Reactivities were then log2-transformed and centred within each sample by adjusting that sample’s median log2 MFI across all beads and adding the array-wide median (approximately 8.2 on the log2 scale), which removes sample-to-sample differences in overall autoantibody reactivity while preserving the original intensity scale; the box-plot axes therefore show median-centred log2 MFI. This per-sample median centring is a data-driven normalisation that expresses each antigen relative to the overall reactivity of the same sample; it is distinct from a correction based on the technical-control beads, and no such correction was applied. The association, survival and competing-risk analyses were performed on the antigen beads, whereas the technical-control beads were used only for quality control. Assay reproducibility was assessed from three reference sera, one healthy control serum and two rheumatic-disease sera with reactivity to the reference proteins (RefPro) included in the array, run in triplicate against all antigens on each of the six assay plates; across the reference-serum reactivities (reference-serum and antigen combinations with a mean MFI below 500 per plate excluded as noise, per the assay quality-control procedure), the median intra-assay coefficient of variation was 4.9 percent and the median inter-assay coefficient of variation was 17.7 percent (Supplementary Table S6). Non-specific binding to the BSA control bead was low (mean 72 ± 73 MFI across samples). All baseline samples were assayed in a single measurement campaign in November 2018 on six assay plates, so no between-campaign batch effect applies; the relevant technical source of variation is between plates, quantified by the median inter-assay coefficient of variation of 17.7 percent given above. Samples were allocated to plates by centre rather than at random. Treatment groups were therefore unevenly distributed across plates (chi-square *p* < 0.001), and the proportion of patients with irAEs also differed between plates (*p* = 0.002), whereas best overall response did not (*p* = 0.28). The leading associations were therefore re-estimated in logistic models adjusted for assay plate as a sensitivity analysis (see Section 3).

### 4.3. Clinical Endpoints

The principal clinical endpoints were best overall response (according to RECIST v1.1) and development of irAEs. For downstream analyses, patients were grouped into responders (CR/PR) and non-responders (SD/PD), respectively. Analyses were performed separately within treatment strata to avoid conflating potentially distinct associations under CTLA-4 and PD-1 blockade. Colitis was additionally explored within the CTLA-4-based population (ipilimumab monotherapy and ipilimumab/nivolumab; 131 patients with known colitis status) because of its clinical relevance and frequency under CTLA-4-antibody-containing regimens. Immune-related adverse events were graded according to CTCAE 4.03 and attributed to therapy by the treating investigator, with events recorded from treatment initiation to the last follow-up. Tumour response was assessed by RECIST version 1.1, with imaging every three months by computed tomography of the chest, abdomen and pelvis at all participating centres. The number of evaluable patients differed between endpoints because of missing data, with best response available for 307 of 309 patients, irAE status for 284, and colitis status for 293, and each analysis is reported against its own denominator. The median follow-up (reverse Kaplan–Meier) was 47 months overall and was substantial in every group (ipilimumab monotherapy 66 months, ipilimumab/nivolumab 34, nivolumab monotherapy 37, pembrolizumab without prior CTLA-4 45, pembrolizumab after ipilimumab 58), exceeding the 68-week median time to colitis in the pembrolizumab group, so that late events could be captured. Follow-up was similar in patients with and without irAEs (47 versus 45 months), indicating that differential observation time is unlikely to have biased the classification into irAE and no-irAE groups; time to irAE was additionally modelled with death as a competing event (Supplementary Figure S9).

### 4.4. Statistical Analysis

For each antigen within each pre-specified treatment group, group differences were assessed using label-permutation testing with 5000 permutations. Significance Analysis of Microarrays (SAM), applied to the same median-centred data, was used only as a sensitivity analysis (see below). Exploratory overall survival (OS) analyses were performed in the pooled cohort and within treatment strata. For each antigen, baseline autoantibody reactivity was dichotomized into high (>mean + 1 standard deviation) and low (≤mean + 1 standard deviation) groups. Survival distributions were estimated using the Kaplan–Meier method and compared using log-rank tests. Hazard ratios (HRs) were calculated with the low-reactivity group as the reference.

A single test was applied consistently to every box plot and volcano plot: a label-permutation test using the difference in group means of the log2 reactivity as the statistic, with 5000 permutations and a two-sided *p*-value. The *p*-values annotated on the figures are these permutation *p*-values. Multiple testing was controlled with the Benjamini–Hochberg procedure, applied separately to the 86 antigens tested for each endpoint in each treatment group, and the resulting false-discovery-rate q-value is reported beside each panel, so that nominal associations are distinguished explicitly from multiplicity-adjusted ones. Because this is a focused discovery panel, most per-antigen associations are nominal and hypothesis-generating; Significance Analysis of Microarrays was retained only as a sensitivity check and gave concordant rankings (Spearman correlation between the SAM and permutation rankings 0.93 to 0.97 across the endpoint and treatment-group analyses). Since a difference in the direction of an association between separate strata does not by itself demonstrate effect modification, regimen dependence was tested formally in logistic models fitted to all patients with the respective endpoint available. Each model contained the marker as a continuous variable (median-centred log2 MFI, so that odds ratios refer to one log2 unit), an indicator for the regimen of interest (coded 1 for that regimen and 0 for all other treatment groups combined, which served as the reference) and the product of the two. The interaction odds ratio is the ratio of the per-log2-unit odds ratio in the regimen of interest to that in all other patients and is reported with its 95% confidence interval. Twelve interaction tests were performed for the leading markers; their *p*-values are not adjusted for multiplicity, and the interaction results are therefore nominal and exploratory (for information, Benjamini–Hochberg adjustment across the twelve tests gave q-values between 0.009 and 0.14). For survival, the cut-point at the mean plus one standard deviation was derived from the pooled cohort rather than within groups; each antibody was additionally modelled as a continuous variable (Cox hazard ratio per log2 unit), and the toxicity endpoints were examined with competing-risk methods that estimate the cumulative incidence of the event, with death treated as a competing risk, complemented by cause-specific Cox models.

Overall survival was measured from treatment initiation with censoring at the last contact. Analyses were performed in Python (version 3.13), and the areas under the ROC curve with their DeLong confidence intervals were computed as implemented in the pROC package [23]. Hazard ratios were estimated by Cox proportional-hazards regression (Newton-Raphson, Breslow handling of ties), and the proportional-hazards assumption was verified for the displayed markers using scaled Schoenfeld residuals correlated with event-time rank (all *p* > 0.2); the data cutoff for survival follow-up was April 2020. The mean plus one standard deviation cut-point was pre-specified to define a high-reactivity subgroup for the Kaplan–Meier display and was computed on the pooled cohort, with continuous modelling serving as the primary analysis; this avoids dichotomising the marker but still assumes proportional hazards and a log-linear relationship between reactivity and the hazard. For overall survival, 344 associations were tested (86 antigens in each of four strata); the curves shown in Figure 8 are those reaching nominal significance in that screen (the pooled anti-PD-L1/CD274 curve, log-rank *p* = 0.050, is shown as borderline), and the complete results, with patient and death counts and 95% confidence intervals for every antigen, are provided in Supplementary Table S2.

Where Significance Analysis of Microarrays (SAM) was used as a sensitivity analysis, it employed a regularised d statistic with the fudge factor set to the median of the antigen-wise standard errors, 2000 label permutations, and a false-discovery-rate q-value estimated, for each antigen, as the mean number of permuted statistics at least as extreme as the observed statistic divided by the number of observed statistics at least as extreme. The complete association results for all 86 antigens, with group sizes, mean differences, permutation *p*-values, Welch t *p*-values and Benjamini–Hochberg q-values, are provided in Supplementary Table S4. All statistical tests were two-sided and were applied consistently across treatment groups and endpoints. Ninety-five percent confidence intervals for the per-antigen mean differences are provided in Supplementary Table S4. For the leading markers, discrimination was summarised by the area under the receiver-operating-characteristic curve with 95 percent confidence intervals by DeLong’s method (as implemented in the pROC package [23]), and the robustness of each association was assessed by resampling, namely the fraction of 2000 bootstrap replicates in which the association kept its direction and nominal significance; these are reported in Supplementary Table S5 and Supplementary Figure S8. The study includes no external validation cohort, so the resampling provides internal robustness only.

## 5. Conclusions

Baseline autoantibodies against cytokines, chemokines, cytokine receptors, and immune-checkpoint proteins showed distinct, exploratory associations with ICI toxicity and response across melanoma treatment regimens. A directionally changing anti-IL17A/anti-CCL2 irAE pattern across CTLA-4- and PD-1-based therapy, anti-IL10 as a leading (exploratory) irAE signal during pembrolizumab without prior CTLA-4 therapy, and regimen-specific response associations nominate a focused set of candidate humoral markers for prospective and functional validation. The data suggest that biomarker development should be regimen-resolved rather than aimed at a universal autoantibody classifier; none of these markers is ready for clinical use.

## Figures and Tables

**Table 1:** Patient characteristics per treatment group. Best response was not documented for 2 patients in the ipilimumab/nivolumab group, so the CR, PR, SD and PD counts in that column sum to 63 rather than 65.

Characteristic	Ipi-Mono	Ipi/Nivo	Nivo-Mono	Pembro- Never-ipi	Pembro- After-ipi	Pembro (All)	All
Patients per treatment group	76	65	19	100	49	149	309
*Patients per institution*							
Heidelberg	58 (76%)	32 (49%)	4 (21%)	38 (38%)	2 (4%)	40 (27%)	134 (43%)
Homburg	18 (24%)	5 (8%)	12 (63%)	16 (16%)	4 (8%)	20 (13%)	55 (18%)
Tübingen	0 (0%)	11 (17%)	0 (0%)	20 (20%)	3 (6%)	23 (15%)	34 (11%)
Zürich	0 (0%)	9 (14%)	1 (5%)	14 (14%)	39 (80%)	53 (36%)	63 (20%)
Dresden	0 (0%)	8 (12%)	2 (11%)	12 (12%)	1 (2%)	13 (9%)	23 (7%)
*Demographics*							
Median age, years (range)	62 (30–86)	58 (29–84)	70 (53–89)	70 (26–92)	63 (30–93)	68 (26–93)	66 (26–93)
*Sex*							
Female	27 (36%)	30 (46%)	8 (42%)	34 (34%)	13 (27%)	47 (32%)	112 (36%)
Male	49 (64%)	35 (54%)	11 (58%)	66 (66%)	36 (73%)	102 (68%)	197 (64%)
*irAE*							
Any irAE, any grade	24 (32%)	31 (48%)	1 (5%)	26 (26%)	8 (16%)	34 (23%)	90 (29%)
irAE grade 3 or 4	1 (1%)	23 (35%)	1 (5%)	11 (11%)	8 (16%)	19 (13%)	44 (14%)
Median time until irAE (weeks)	7	6	23	27	28	27	10
*ir-colitis*							
Colitis, any grade	12 (16%)	14 (22%)	0 (0%)	8 (8%)	3 (6%)	11 (7%)	37 (12%)
Colitis grade 3 or 4	1 (1%)	8 (12%)	0 (0%)	1 (1%)	2 (4%)	3 (2%)	12 (4%)
Median time until colitis (weeks)	6	5	NA	68	12	24	8
*Best response (RECIST)*							
CR	5 (7%)	4 (6%)	5 (26%)	25 (25%)	10 (20%)	35 (23%)	49 (16%)
PR	15 (20%)	15 (23%)	1 (5%)	24 (24%)	11 (22%)	35 (23%)	66 (21%)
SD	10 (13%)	10 (15%)	4 (21%)	15 (15%)	9 (18%)	24 (16%)	48 (16%)
PD	46 (61%)	34 (52%)	9 (47%)	36 (36%)	19 (39%)	55 (37%)	144 (47%)
ORR (CR + PR)	20 (26%)	19 (29%)	6 (32%)	49 (49%)	21 (43%)	70 (47%)	115 (37%)
DCR (CR + PR + SD)	30 (39%)	29 (45%)	10 (53%)	64 (64%)	30 (61%)	94 (63%)	163 (53%)
Median follow-up, months (reverse-KM)	66	34	37	45	58	48	47
Elevated LDH (>ULN), n/available	12/17	26/53	6/16	17/63	10/46	27/109	71/195
Karnofsky PS high, n/available	15/18	48/53	16/16	60/63	46/47	106/110	185/197
BRAF V600-mutant, n/assessable	7/17	18/49	2/15	19/62	15/47	34/109	61/190

## Data Availability

De-identified antigen-level reactivity data and the associated clinical variables are available from the corresponding author on reasonable request, subject to the ethics approvals and data-protection agreements of the participating centres. The analytical code used for the association, survival, and competing-risk analyses is available from the corresponding author on reasonable request.

## Supplementary Materials

The following supporting information can be downloaded at: https://www.mdpi.com/article/10.3390/ijms27219746/s1.
